# Self-Assembly of Human Embryonic-Stem-Cell-Derived Keratinocytes and Fibroblasts into 3D Spheroid Structures for Epidermal Regeneration In Vivo

**DOI:** 10.3390/cells15070631

**Published:** 2026-03-31

**Authors:** Chenghao Cai, Huan Liu, Shuwen Liu, Ziyue Zhao, Pengqin Xu, Yiran Wang, Jialiang Wang, Aobuliaximu Yakupu, Jiaming Shao, Miao Pan, Wei Zhang, Chunmao Han, Fang He, Lina Dong, Xingang Wang

**Affiliations:** 1 Department of Burns & Wound Care Center, The Second Affiliated Hospital of Zhejiang University School of Medicine, Hangzhou 310009, China; 12418365@zju.edu.cn (C.C.);; 2 Department of Plastic Surgery, The Second Affiliated Hospital of Zhejiang University School of Medicine, Hangzhou 310009, China

**Keywords:** human embryonic stem cells, keratinocytes, epidermal reconstruction

## Abstract

Introduction: Extensive thermal injury remains a formidable clinical challenge, primarily due to the profound deficit of autologous donor skin, which necessitates prolonged hospitalization and escalates healthcare expenditures. While human embryonic stem cells (hESCs) offer a theoretically inexhaustible source for regenerative therapy, optimizing their differentiation and engraftment remains critical for clinical translation. Methods: We used a three-stage protocol to induce the differentiation of hESCs into keratinocytes (KCs). To optimize the delivery of hESC-derived keratinocytes (EKCs), human dermal fibroblasts (HFBs) were utilized to provide essential extracellular matrix (ECM) and microenvironmental support. The two cell types could self-assemble into 3D spheroids. After optimizing the size and cell proportion, these spheroids were subsequently transplanted onto full-thickness dorsal wounds in immunodeficient mice to evaluate their regenerative capacity. Results: hESC-derived keratinocytes exhibited the expression of stage-specific epidermal markers, confirming high differentiation efficiency. In vitro, EKCs demonstrate the capacity to form stratified epidermal structures. By self-assembling into spheres with dermal fibroblasts, the EKCs demonstrated successful engraftment and sustained survival in vivo. The transplantation of these 3D spheroids significantly accelerated wound closure and re-epithelialization compared with controls. Conclusions: This study establishes a robust cell therapy approach characterized by a short preparation cycle with high differentiation efficiency and high transplantation survival rate, offering a novel strategy for the treatment of extensive skin defects.

## 1. Introduction

Large-area thermal burns, diabetic foot ulcers, and pressure sores represent significant global medical and economic burdens, with burn injuries alone affecting over 100 million people annually [1]. Timely and effective wound closure is critical for saving lives and improving clinical outcomes. Currently, autologous skin grafting remains the gold standard for treating extensive skin loss [2]. However, this approach is constrained by the scarcity of autologous donor sites, the risk of secondary morbidity, and the development of hypertrophic scarring and contracture [3]. These limitations often result in profound functional impairment and psychological distress, severely diminishing the patient’s quality of life. Furthermore, the supply–demand imbalance for donor skin becomes increasingly acute as the total body surface area of the wound expands [4]. While allografts and cultured epithelial autografts are clinically available [5,6], they remain hindered by limited sources and prolonged cultivation periods. Consequently, there is an urgent need in regenerative medicine to develop a novel therapeutic modality that transcends donor limitations, possesses superior biocompatibility, and rapidly reconstructs the skin barrier.

In this context, cell-based therapies leveraging the robust proliferative and differentiative capacities of stem cells offer a revolutionary paradigm for skin regeneration [7]. The core advantage lies in the ability to construct active skin tissues in vitro from “seed cells,” facilitating true “regeneration” rather than mere “repair”. This strategy not only addresses the fundamental shortage of skin sources but also modulates the wound microenvironment by secreting essential growth factors [8], thereby promoting integration between the graft and the host. Human embryonic stem cells (hESCs), as pluripotent entities, exhibit unparalleled potential as a seed cell source due to their near-infinite self-renewal and the capacity for large-scale industrial expansion [9,10]. Most importantly, by precisely manipulating key developmental signaling pathways—including transforming growth factor-beta (TGF-β), fibroblast growth factor (FGF), and bone morphogenetic proteins (BMPs)—hESCs can be efficiently induced into core skin components such as keratinocytes and fibroblasts [11,12,13]. Recent breakthroughs further demonstrate that skin organoids derived from hESCs can recapitulate complex stratified epidermal structures and even functional appendages like hair follicles and glands, marking a fundamental shift toward full-thickness tissue regeneration [1].

However, the clinical translational efficacy of seed cells is heavily dictated by the mode of transplantation. Traditional cell suspensions are prone to significant cell loss and suffer from poor control over delivery efficiency [14]; moreover, cells often undergo massive apoptosis due to abrupt changes in osmotic pressure and the microenvironment upon leaving the culture medium. Although cell sheet technology preserves the endogenous extracellular matrix (ECM) and enhances survival [15,16], it requires specialized culture conditions, is time-intensive, and results in fragile constructs that complicate surgical handling. While current bio-scaffolds provide physical support, their degradation rates are often asynchronously matched with tissue integration, and natural matrices like Matrigel pose risks of xenogenicity and batch variability [17]. Consequently, many tissue-engineered skins rely predominantly on paracrine effects for therapeutic efficacy [18]. Recent findings suggest that 3D cell aggregates or spheroids can significantly enhance juxtacrine and paracrine signaling by mimicking the in vivo niche [19]. In native physiology, human dermal fibroblasts (HFBs) secrete abundant collagen and ECM to provide mechanical support and biochemical cues to the epidermis [20]. Evidence indicates that grafts lacking mesenchymal–ectodermal signaling (e.g., Gibbin-dependent signals) result in incomplete epidermal maturation and stratification [21]. Based on this, we propose a synergistic strategy: co-culturing HFBs and hESC-derived keratinocytes (EKCs) to form composite spheroids and utilizing HFBs to establish an in situ ECM network that provides immediate physiological support and protection. This 3D synergistic model not only resists the impact of the hostile wound microenvironment to prevent cell death but also promotes high-quality integration and stratification by mimicking epithelial–mesenchymal interactions (EMI) [22].

**Figure 1:**
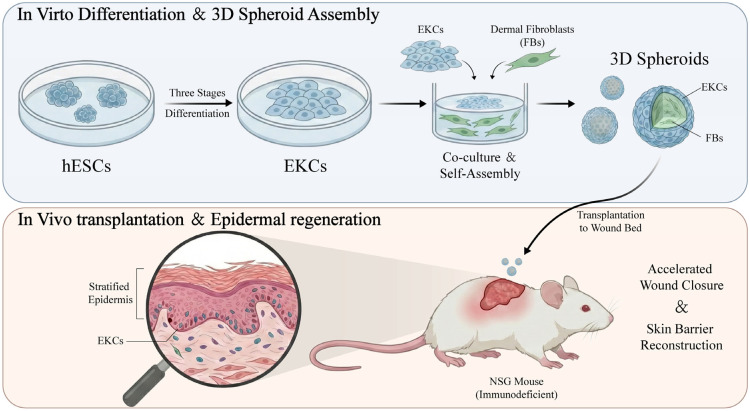
Schematic of hESC-derived keratinocytes (EKCs) differentiation, transplantation, and promotion of wound closure.

In this study, we first achieved highly efficient directed differentiation of hESCs into functional keratinocytes, which were subsequently co-cultured with human dermal fibroblasts to self-assemble into highly active composite spheroids. By transplanting these spheroids onto full-thickness skin defect wounds in immunodeficient mice, we systematically analyzed their performance in terms of wound colonization, epidermal stratification, and regenerative healing (Figure 1). Our results demonstrate that this hESC-based 3D spheroid strategy significantly accelerates wound closure and exhibits unique advantages in reconstructing the skin barrier. This study establishes a novel cell therapy pathway characterized by a short preparation cycle, standardized large-scale manufacturing, and ease of surgical operation, thereby facilitating the clinical application of regenerative medicine in skin tissue engineering.

## 2. Materials and Methods

### 2.1. hESC Culture and EKCs Differentiation

hESC Culture: H9 human embryonic stem cells were provided by the Department of Burn Surgery at the Second Affiliated Hospital of Zhejiang University School of Medicine through a Material Transfer Agreement (MTA) with Yuansheng Biotechnology Co., Ltd. (Hangzhou, China). Human embryonic stem cells were maintained on Matrigel-coated (Corning, Corning, NY, USA) plates in mTeSR Plus medium (STEMCELL Technologies, Vancouver, BC, Canada) with daily medium changes. Cells were passaged at an appropriate density using ReLeSR dissociation reagent (STEMCELL Technologies, Vancouver, BC, Canada) and re-plated in mTeSR Plus medium supplemented with 10 μM Y27632 (STEMCELL Technologies, Vancouver, BC, Canada).

EKCs Differentiation: A sequential three-stage induction protocol was employed. Stage 1: Cells were cultured in Defined Keratinocyte-SFM (Thermo Fisher, Waltham, MA, USA) supplemented with 0.3 μg/mL retinoic acid (Sigma-Aldrich, St. Louis, MO, USA) and 25 ng/mL BMP4 (R&D Systems, Minneapolis, MN, USA) for 5 days. Stage 2: The medium was switched to CnT-07 (CELLnTEC, Bern, Switzerland) supplemented with 20 ng/mL EGF (Sigma-Aldrich, St. Louis, MO, USA) for another 5 days. Stage 3: Cells were maintained in CnT-07 supplemented with 10 μM Y27632 for 5 days or longer. During Stage 1 to 3, if cell density became excessive, cells were passaged onto plates pre-coated with 15 μg/mL Type IV Collagen (Sigma-Aldrich, St. Louis, MO, USA) to select for epidermal lineage commitment. Culture continued in Stage 3 medium until cells exhibited classic cobblestone-like keratinocyte morphology.

### 2.2. Human Dermal Fibroblasts Culture

Human dermal fibroblasts were obtained from Procell Life Science & Technology Co., Ltd. (Wuhan, China; Cat. CP-H103Y). The cells were cultured in Dulbecco’s Modified Eagle Medium (DMEM, high glucose; Gibco, Grand Island, NY, USA) supplemented with 10% fetal bovine serum (Sigma-Aldrich, St. Louis, MO, USA). Upon reaching approximately 70% confluence, the cells were passaged using 0.25% trypsin-EDTA (Gibco, Grand Island, NY, USA) for passaging or subsequent experiments.

### 2.3. Isolation and Culture of Primary Human Epidermal Keratinocytes

Primary human epidermal keratinocytes were isolated from foreskin obtained from healthy child donors, following informed consent and approval by the Institutional Ethics Committee. Subcutaneous adipose tissue was carefully removed, and the skin was cut into small pieces. The skin fragments were then incubated in a 0.2% dispase II (Solarbio, Beijing, China) solution overnight at 4 °C. Following dispase treatment, the epidermal layer was carefully peeled away from the dermis and subsequently incubated in 0.25% trypsin-EDTA (Gibco, Grand Island, NY, USA) for 15 min at 37 °C. Trypsin activity was neutralized by adding an equal volume of DMEM (Gibco, Grand Island, NY, USA) supplemented with 10% FBS (Sigma-Aldrich, St. Louis, MO, USA). The cell suspension was filtered through a 70 µm cell strainer to remove stratum corneum debris and then centrifuged and cultured in EpiLife medium (Thermo Fisher, Waltham, MA, USA). The culture medium was refreshed every 2 days until cells reached 70% confluence for passaging or subsequent experiments.

### 2.4. Trilineage Differentiation Assay

The pluripotency of hESCs was validated using the STEMdiff™ Trilineage Differentiation Kit (STEMCELL Technologies, Vancouver, BC, Canada). hESCs were seeded at specific densities and induced into three germ layers according to the manufacturer’s instructions. Mesoderm and endoderm differentiation were completed within 5 days, while ectoderm induction required 7 days. Samples were collected at corresponding time points for characterization.

### 2.5. Quantitative RT-PCR

Total RNA was extracted using an RNA extraction kit (Accurate Biology, Changsha, China) and reverse-transcribed into cDNA using the Evo M-MLV RT Kit (Accurate Biology, Changsha, China). Real-time PCR was performed using SYBR Green Pro Taq HS Premix (Accurate Biology, Changsha, China). Relative gene expression was calculated using the 2^−ΔΔCT^ method, with GAPDH or RPS18 serving as the internal reference gene.

### 2.6. Immunofluorescence Staining

Cells or frozen tissue sections were fixed in 4% paraformaldehyde for 15 min, followed by permeabilization and blocking using specialized reagents (Beyotime, Shanghai, China). Samples were incubated overnight at 4 °C with primary antibodies, including anti-K5, anti-K14, anti-K10, and anti-human nuclei (AHN) antibody (all from Abcam, Cambridge, United Kingdom) at manufacturer-recommended dilutions. After three PBS washes, secondary antibodies (Abcam, Cambridge, United Kingdom) were applied at 37 °C for 50 min. Nuclei were counterstained with DAPI (Solarbio, Beijing, China) for 10 min prior to mounting and imaging.

### 2.7. In Vitro Construction of 3D Skin Models via ALI Culture

To evaluate the stratification capacity of EKCs, second-passage EKCs were seeded onto Type IV Collagen-coated Transwell inserts (0.4 μm pore size; Corning, Corning, NY, USA). Upon reaching confluence, the medium in the upper chamber was removed to create an air–liquid interface. The lower chamber was supplied with CnT-07 medium containing 1.5 mM Ca^2+^ to induce terminal differentiation. The medium was refreshed every two days until a stratified epidermal structure formed.

### 2.8. Transmission Electron Microscopy

Epidermal tissues harvested after 3–4 weeks of ALI culture were pre-fixed in 2.5% glutaraldehyde for 2 h and post-fixed in 1% osmium tetroxide for 2 h. Samples were dehydrated through a graded ethanol series (50–100%) and embedded in epoxy resin. Ultrathin sections (70 nm) were prepared and double-stained with uranyl acetate and lead citrate. Images were captured using a transmission electron microscope (Thermo Fisher, Waltham, MA, USA) to observe intercellular desmosomes.

### 2.9. Proliferation Kinetics: Population Doubling Time

The proliferative capacity of EKCs was assessed by seeding 4 × 10^5^ cells in 6-well plates. Cells were harvested and counted every 4 days and re-seeded at the initial density until growth significantly declined. PDT and the number of divisions per generation (n) were calculated using the following formulas: PDT = 4 × log_e_(2)/log_e_(Total cell number/400,000) and n = 4/PDT.

### 2.10. Flow Cytometry

Preparation of EKC single-cell suspensions was performed. Cells were washed twice with cold Stain Buffer (BD, Franklin Lakes, NJ, USA) and pelleted by centrifugation. Fixation and permeabilization of EKCs were carried out using a Fixation/Permeabilization Kit (BD, Franklin Lakes, NJ, USA) according to the manufacturer’s instructions. Following fixation, cells were washed twice with Stain Buffer. Fluorescent antibodies (Abcam, Cambridge, United Kingdom) were diluted to predetermined optimal concentrations in Stain Buffer and added to the tubes containing the cell suspensions. The mixtures were incubated for 20 min on ice, protected from light. After incubation, cells were washed twice, and the resulting cell pellets were resuspended in 200 µL of Stain Buffer. Stained cells were then transferred from microwell plates to appropriate tubes for flow cytometric analysis. Finally, samples were analyzed using a flow cytometer (BD, Franklin Lakes, NJ, USA).

### 2.11. Construction and Analysis of 3D Cell Spheres

EKCs and HFBs were mixed at different ratios and seeded into a low-adhesion U-bottom 96-well plate (Corning, Corning, NY, USA). The plate was centrifuged at 1000 rpm for 5 min. After 24 h of incubation, the two cell types self-assembled into heterotypic cell spheroids. For pathological analysis of the 3D spheroids, co-cultured spheroids were harvested on day 5, embedded in OCT compound, and sectioned using a cryostat (Leica, Wetzlar, Germany). Sections were processed for H&E staining and immunofluorescence to analyze internal structural organization. During the culture period, cell spheroids can be harvested at any time for live/dead staining. First, retrieve the samples and wash them with PBS twice to remove any residual culture medium solution. Then, add the prepared PI working solution (EFL-Tech, Suzhou, China) and incubate at room temperature for 10 min. After removing the PI working solution, gently wash once with an adequate amount of PBS and discard the supernatant. Next, add a sufficient amount of the prepared AM working solution (EFL-Tech, Suzhou, China) and incubate at room temperature for 20 min. Following the removal of the AM working solution, perform another gentle wash with PBS. Finally, observe the live/dead staining results of the cells under a microscope.

### 2.12. Animal Model and Transplantation

Six-week-old male NSG immunodeficient mice (Shanghai Model Organisms Center, Shanghai, Shanghai, China) were used in accordance with ethical guidelines. Mice were anesthetized via intraperitoneal injection of 0.3% pentobarbital sodium. After disinfection with iodophor, full-thickness skin defects (1 cm diameter) were surgically created on the dorsal skin. All cell treatment groups received the same number of cell spheres transplanted onto the wound surface, while the blank control group was treated with 100 microliters of culture medium. The wounds were secured with hydrogel dressings, hydrocolloid dressings, and elastic bandages, which were changed every 3 days while maintaining a moist environment. Tissues were harvested at different time points post-transplantation.

### 2.13. Histological Analysis (H&E Staining)

Animal tissues were fixed in 4% PFA, dehydrated, and embedded in paraffin. Sections were stained using an H&E staining kit (Solarbio, Beijing, China) according to the manufacturer’s protocol. Morphological changes and re-epithelialization were observed under a light microscope (Leica, Wetzlar, Germany).

### 2.14. Statistical Analysis

The GraphPad Prism software (version 10.0, Boston, MA, USA) was utilized for statistical analysis. All data were first tested for normality using the Shapiro–Wilk test to confirm whether the data met the assumptions of parametric testing. For comparisons between two groups, an independent-samples *t*-test was used. For comparisons among multiple groups, a one-way analysis of variance was performed, followed by Tukey’s test for post hoc multiple comparisons. For data involving different time points, a two-way analysis of variance was employed to evaluate the interaction effect between the treatment factor and the time factor, and multiple comparisons were conducted using Tukey’s test. Data are presented as mean ± standard deviation. A significance level of *p* < 0.05 was considered statistically significant (* *p* < 0.05, ** *p* < 0.01, *** *p* < 0.001).

## 3. Results

### 3.1. In Vitro Differentiation of Human Embryonic Stem Cells into the Epidermal Lineage

**Figure 2:**
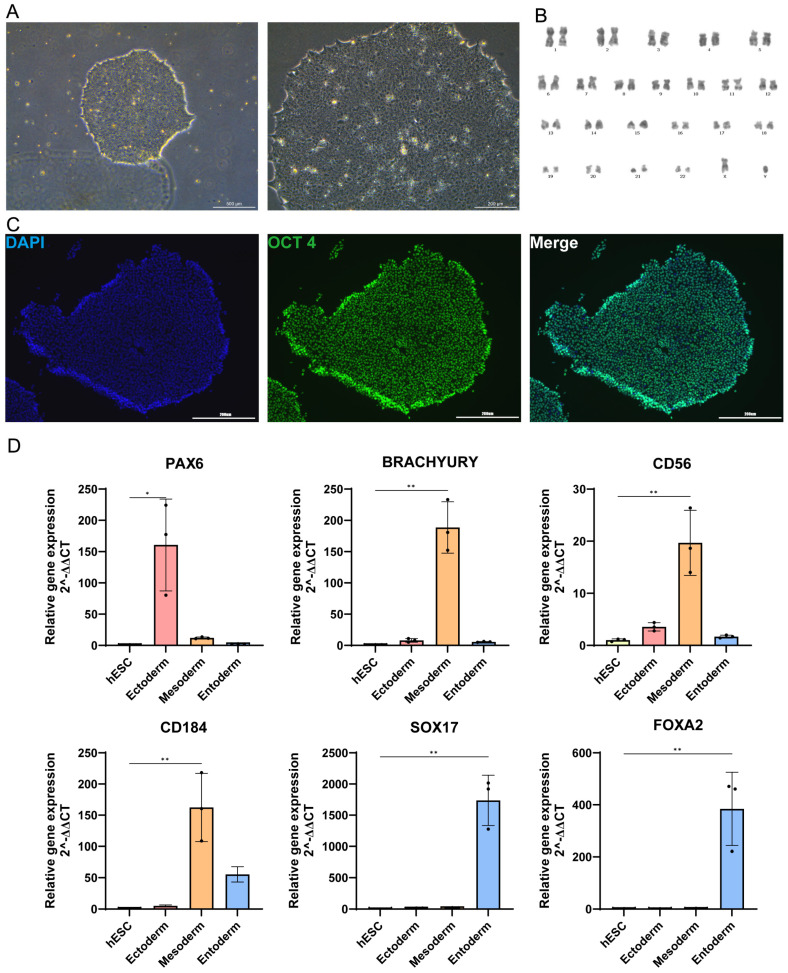
Culture and characterization of human embryonic stem cells (hESCs). (**A**) Representative bright-field microscopy image of hESCs exhibiting typical colony morphology. Scale bar = 500 μm (**upper**), 200 μm (**lower**). (**B**) Karyotype analysis of hESCs. (**C**) Immunofluorescence staining of the pluripotency marker OCT4 in hESCs. Scale bar = 200 μm. (**D**) RT-qPCR analysis of marker gene expression following in vitro trilineage differentiation of hESCs into the three germ layers (ectoderm, mesoderm and endoderm). (n = 3 independent samples per group. Data are presented as mean ± SD,* *p* < 0.05, ** *p* < 0.01).

To induce keratinocyte differentiation, we first characterized the status of human embryonic stem cells. Undifferentiated hESCs exhibited typical colony morphology with tight intercellular junctions and well-defined borders, showing no signs of spontaneous differentiation at the colony periphery (Figure 2A, left panel). Under high magnification, the cells displayed distinct nuclei and a high nuclear-to-cytoplasmic ratio (Figure 2A, right panel). Karyotype analysis confirmed that the hESCs maintained a stable human diploid chromosomal complement even after extensive propagation, ensuring genomic integrity during long-term culture (Figure 2B). Pluripotency was validated by immunofluorescence staining, which showed high expression of the key transcription factor OCT4 (Figure 2C). Furthermore, the trilineage differentiation potential was confirmed by inducing hESCs to form three germ layers: the ectoderm (marked by *PAX6*), the mesoderm (marked by *Brachyury*, *CD56*, and *CD184*), and the endoderm (marked by *SOX17* and *FOXA2*) (Figure 2D).

**Figure 3:**
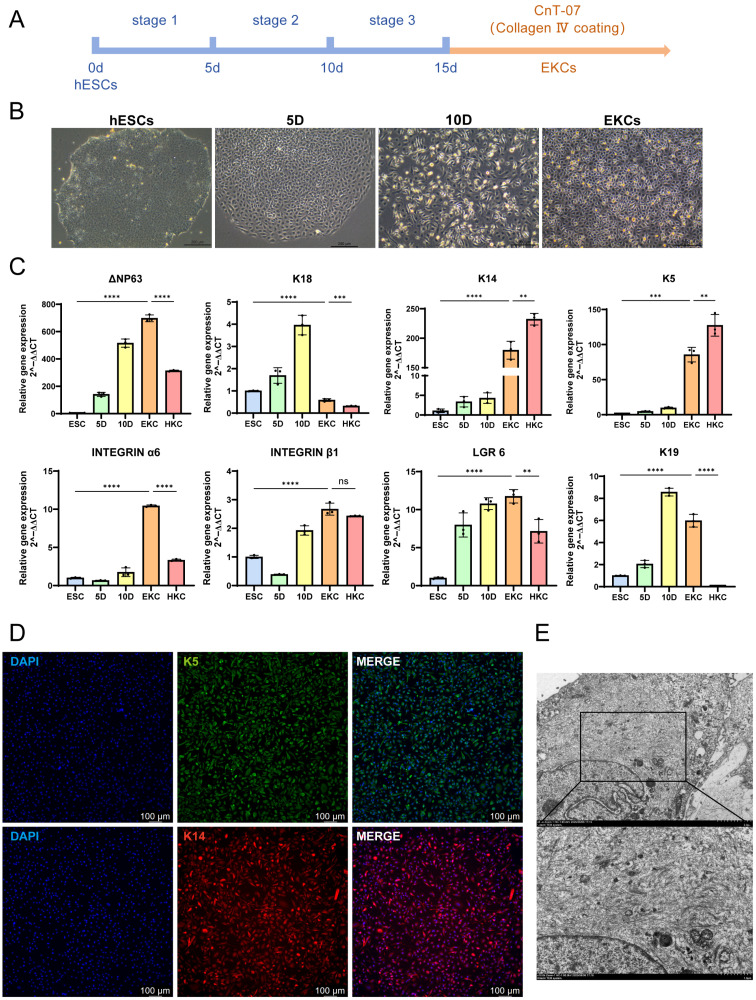
Induction and characterization of EKCs. (**A**) Schematic representation of the three-stage induction protocol for EKCs differentiation. (**B**) Representative bright-field microscopy images showing EKCs morphology at indicated time points during the differentiation process. Scale bar = 200 μm. (**C**) Expression profiles of hallmark genes at various time points during EKCs induction and differentiation. (n = 3 independent samples per group. Data are presented as mean ± SD, ** *p* < 0.01, *** *p* < 0.001, **** *p* < 0.0001, ns: not significant.) (**D**) Immunofluorescence staining of classic keratinocyte markers in successfully induced EKCs. Scale bar = 50 μm. (**E**) Representative transmission electron microscopy images demonstrating the expression of keratin intermediate filaments in EKCs. Scale bar = 2 μm (**upper**), 1 μm (**lower**).

**Figure A1:**
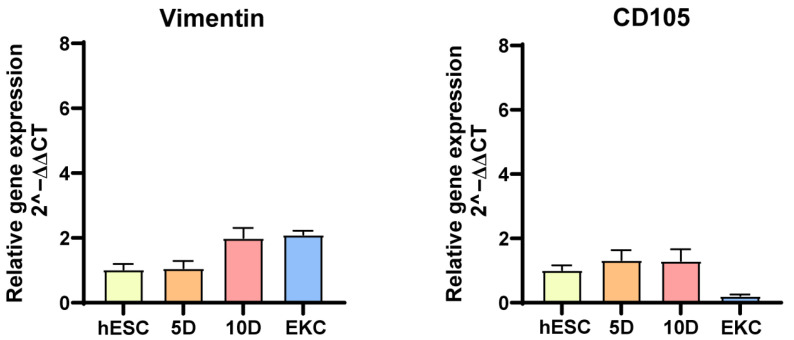
The expression of the fibroblast marker gene vimentin and the mesenchymal stem cell marker gene CD105 during the EKCS differentiation process. (n = 3 independent samples per group. Data are presented as mean ± SD).

Following this, a three-stage induction protocol was executed to direct differentiation (Figure 3A). Morphological changes were observed progressively, with keratinocyte-like clones exhibiting typical “cobblestone” morphology appearing between days 15 and 20 (Figure 3B). The resulting cells were then subcultured onto type IV collagen-coated plates to mimic the basement membrane environment and selectively enrich for epidermal progenitors, as only successfully differentiated cells could effectively adhere to the matrix. Gene expression analysis during the differentiation process revealed that *ΔNp63* (an isoform of *TP63*), the earliest marker of the epidermal lineage, was strongly expressed as early as day 5 and remained highly expressed throughout the process. *Keratin 18 (K18)*, a marker for simple monolayer epithelia, was expressed in the early stages and subsequently declined as maturation progressed. The emergence of basal keratinocyte markers, *keratin 5 (K5)* and *keratin 14 (K14)*, in the mid-to-late stages signified the successful commitment of hESCs to the keratinocyte lineage. Additionally, the expression of epidermal stem cell markers *integrin α6*, *integrin β1*, *LGR6* and *keratin 19 (K19)* indicated that the derived keratinocytes retained stemness properties (Figure 3C). The absence of fibroblast contamination—a common issue in such protocols—was confirmed by the negligible expression of vimentin (Figure A1). Further protein-level verification via immunofluorescence staining showed robust expression of K5 and K14 in the derived EKCs (Figure 3D). Furthermore, transmission electron microscopy (TEM) revealed the presence of dense keratin intermediate filaments within the cells, further indicating a highly differentiated state and suggesting that the cells possess the necessary mechanical integrity characteristic of mature epithelial cells (Figure 3E).

### 3.2. EKCs Demonstrate the Capacity to Form Stratified Epidermal Structures In Vitro

**Figure 4:**
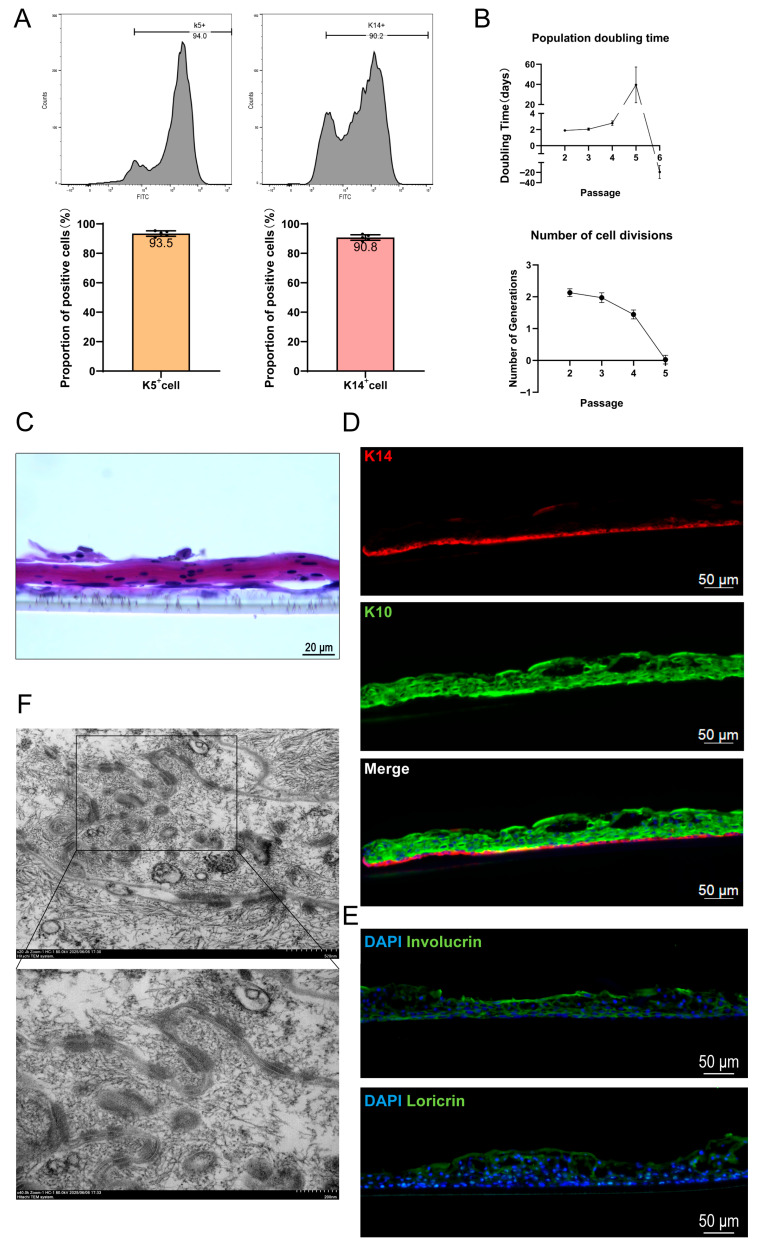
Assessment of EKC differentiation efficiency and in vitro epidermal stratification capacity. (**A**) Evaluation of differentiation efficiency via flow cytometric analysis of K5- and K14-positive cell populations in second-passage EKCs. (n = 5 independent samples per group. Data are presented as mean ± SD) (**B**) Proliferation kinetics of EKCs, showing population doubling time (PDT) and the number of cell divisions per generation. (n = 6 independent samples per group. Data are presented as mean ± SD) (**C**) Representative H&E staining image of the stratified epidermal tissue constructed by EKCs after 20 days of air-liquid interface (ALI) culture. Scale bar = 20 μm. (**D**) Representative immunofluorescence staining images of the stratified epidermal tissue after 20 days of ALI culture. Scale bar = 50 μm. (**E**) Immunofluorescence staining for maturation markers in the stratified epidermal tissue. Scale bar = 50 μm. (**F**) Representative TEM images of the stratified epidermal tissue, demonstrating the formation of mature desmosomes. Scale bar = 500 nm (**upper**), 200 nm (**lower**).

To achieve functional epidermal reconstruction in vivo, it is imperative to obtain a sufficient supply of seed cells that retain native physiological properties. Therefore, we first evaluated the induction efficiency and proliferative capacity of the hESC-derived keratinocytes. K5 and K14 were employed as hallmark markers to assess the efficiency of directed differentiation. Flow cytometric analysis revealed that in second-passage EKCs (defined as P2, where cells from stages 1–3 are designated as P0 and cells at the completion of stage 3 as P1), the average proportions of K5-positive and K14-positive cells reached 93.5% and 90.8%, respectively (Figure 4A). The proliferative potential of EKCs was assessed using the methodology described by Ruiz-Torres et al. [23]. Measurements of population doubling time (PDT) and division frequency demonstrated that hESC-derived EKCs maintained robust proliferative capacity through at least four passages (Figure 4B). Subsequently, we investigated whether these induced EKCs possessed the capacity to form a multilayered structure under appropriate conditions, which is a prerequisite for the construction of functionalized skin in vivo. By utilizing Transwell inserts to establish an air–liquid interface (ALI) culture—a system that mimics the physiological environment and triggers keratinocyte maturation [24]—we observed the formation of a multilayered epidermal structure via hematoxylin and eosin (H&E) staining after approximately 20 days of differentiation (Figure 4C). Immunofluorescence analysis confirmed that K14 was highly expressed in the basal layer, while Keratin 10 (K10) was localized to the suprabasal layers (Figure 4D). This spatial distribution is highly consistent with the architecture of native human epidermis. To further characterize the epidermal constructs, immunostaining was performed for involucrin and loricrin, which are established markers of terminally differentiated keratinocytes. Positive immunoreactivity for both markers was observed throughout the stratified epidermis formed by EKCs (Figure 4E). Desmosomes, essential intercellular junctions that ensure mechanical stability and tight cohesion between keratinocytes [25], were clearly identified within the stratified EKC layers via TEM (Figure 4F). These findings further substantiate that EKCs functionally resemble primary keratinocytes.

### 3.3. EKCs Self-Assemble with HFBs into 3D Spheroids and Acquire a Favorable Support

**Figure 5:**
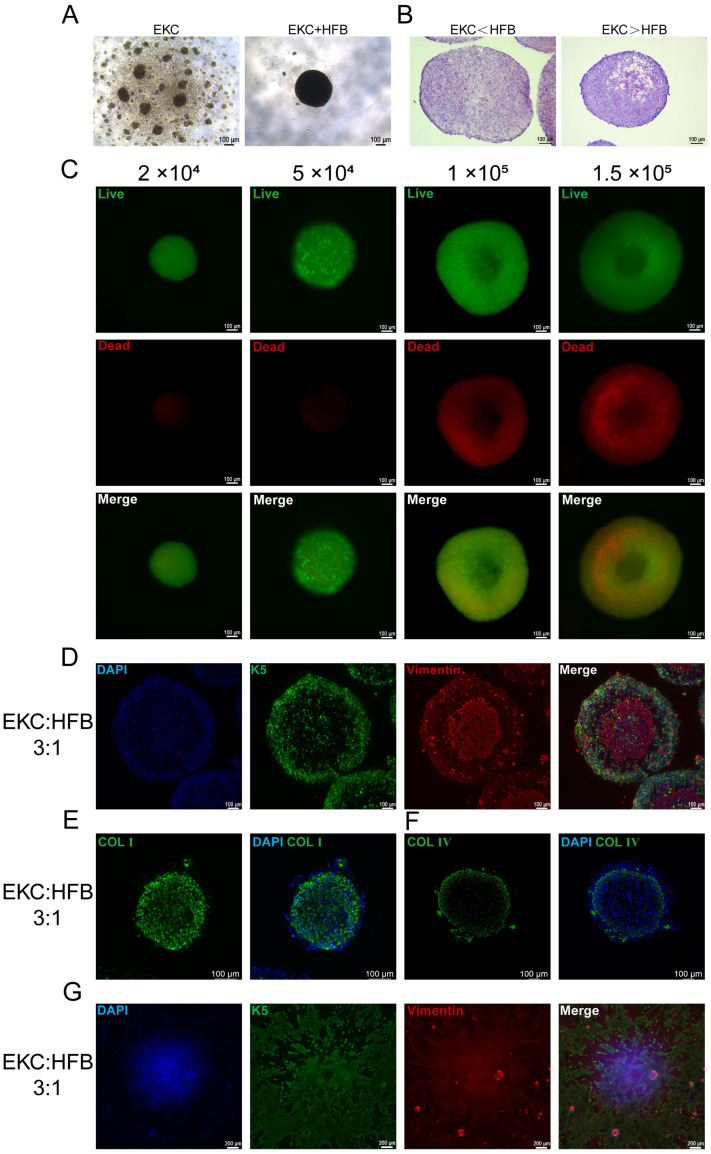
Construction and characterization of EKC–HFB co-culture spheroids. (**A**) Bright-field microscopy images of spheroids formed by EKCs alone (**left**) and EKC–HFB co-culture (**right**) under suspension culture conditions. Scale bar = 100 μm. (**B**) Representative H&E staining images of co-culture spheroids assembled at various EKC-to-HFB ratios. Scale bar = 100 μm. (**C**) Representative live/dead staining images of HFB spheroids formed with varying initial cell numbers after 72 h of culture. Scale bar = 100 μm. (**D**) Representative immunofluorescence images of K5 and Vimentin staining in co-culture spheroids at a 3:1 EKC-to-HFB ratio; each spheroid consists of a total of 50,000 cells. Scale bar = 100 μm. (**E**) Representative immunofluorescence staining of Type I collagen in co-culture spheroids assembled at a 3:1 EKC-to-HFB ratio. Scale bar = 100 μm. (**F**) Representative immunofluorescence staining of Type IV collagen in co-culture spheroids assembled at a 3:1 EKC-to-HFB ratio. Scale bar = 100 μm. (**G**) Representative immunofluorescence staining images of EKC–HFB co-culture spheroids after 24 h of re-plating onto surfaces in vitro. Scale bar = 200 μm.

**Figure A2:**
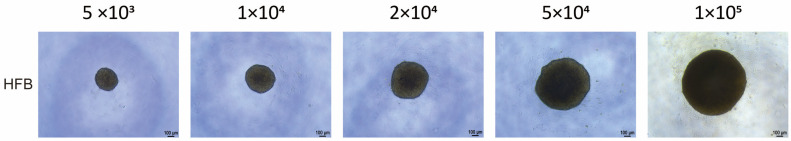
Representative bright-field images of spheroids composed of varying numbers of human dermal fibroblasts after 24 h of cultivation. Scale bar = 100 μm.

**Figure A3:**
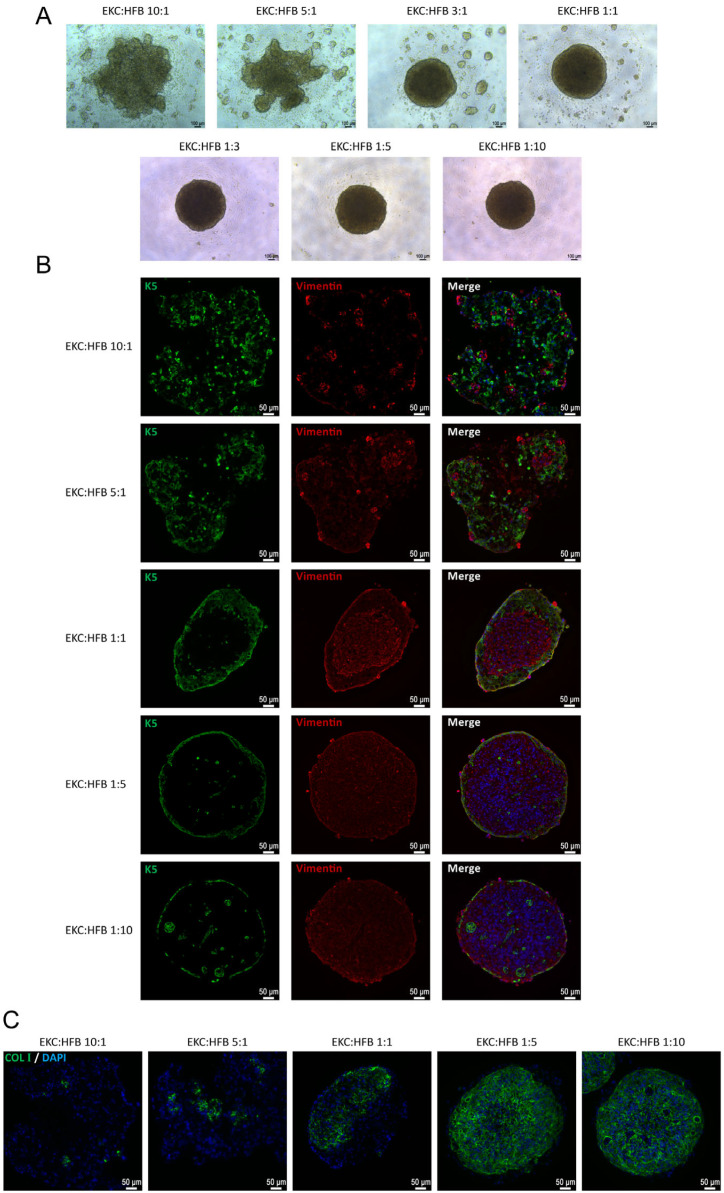
Optimization of the mixing ratio between EKCs and HFBs for spheroid formation. (**A**) Representative bright-field images of self-assembled spheroids composed of EKCs and HFBs at various ratios after 24 h of culture; each spheroid consists of a total of 50,000 cells. Scale bar = 100 μm. (**B**) Representative immunofluorescence images of K5 and Vimentin staining in spheroids at different mixing ratios; each spheroid consists of a total of 50,000 cells. Scale bar = 50 μm. (**C**) Representative immunofluorescence images of Type I collagen staining in spheroids at different mixing ratios; each spheroid consists of a total of 50,000 cells. Scale bar = 50 μm.

In vitro cultured cells are highly sensitive to their external environment; once removed from the liquid medium, they often undergo rapid cell death due to osmotic pressure fluctuations. Therefore, to ensure the successful engraftment of hESC-derived keratinocytes in vivo, we aimed to provide a supportive microenvironment. In native skin, dermal fibroblasts secrete abundant ECM, which provides critical structural support and a functional microenvironment for the growth and migration of epidermal cells [26]. To replicate this, we introduced human dermal fibroblasts to supply the necessary ECM for EKCs. Our investigation revealed that pure EKCs could not form dense aggregates under serum-free conditions. However, in the presence of HFBs, the two cell types spontaneously self-assembled into 3D spheroids (Figure 5A). The mixing ratio of EKCs to HFBs was directly correlated with the compactness of the spheroids. A higher proportion of fibroblasts was correlated with tighter cell arrangement and enhanced mechanical integrity of the spheroids. Conversely, an increasing ratio of EKCs was correlated with larger intercellular spaces and a more fragile spheroid structure (Figure 5B). We attribute these findings to the superior ECM-secreting capacity of fibroblasts compared to EKCs. To optimize parameters for subsequent transplantation, we screened both the spheroid size and cell ratios. Given that fibroblast interaction drives spheroid formation, we initially utilized pure HFBs to determine the ideal size (Figure A2). To avoid central necrosis caused by nutrient deprivation in excessively large spheroids, we performed Live/Dead staining. Results indicated that spheroids containing 100,000 and 150,000 cells exhibited substantial apoptosis in the core by 72 h and 96 h, whereas those composed of 50,000 and 20,000 cells maintained high viability (Figure 5C). In order to ensure the efficiency of cell transplantation, a total of 50,000 cells per spheroid was selected for further experiments. Subsequently, the ratio of EKCs to HFBs within the 50,000-cell spheroids was systematically optimized. When mixing EKCs and HFBs at various ratios, we observed that insufficient fibroblast proportions failed to produce stable spheroids, which often fragmented during handling (Figure A3A). To balance spheroid stability with a maximized EKC proportion, we ultimately established a maximum EKC-to-HFB ratio of 3:1. To maximize the therapeutic potential for transplantation, we selected the 3:1 ratio to provide the highest possible number of EKCs while maintaining structural stability. Co-immunofluorescence staining for K5 and vimentin revealed a characteristic architecture: a continuous EKC layer on the periphery and aggregated HFBs in the core (Figure A3B and Figure 5D). Immunofluorescence staining corroborated the aforementioned finding, demonstrating that a high proportion of EKCs leads to spheroid disintegration. Type I collagen secreted by HFBs was detected throughout the spheroids, acting as a scaffold that integrated the cells and provided an essential extracellular matrix for the EKCs (Figure A3C and Figure 5E). Notably, type IV collagen tended to localize at the boundary between EKCs and HFBs (Figure 5F), mirroring the basement membrane structure of native skin. To confirm that the cells could interact with the wound bed after transplantation, we performed in vitro spreading assays using a 3:1 ratio. Fluorescence staining demonstrated that both cell types could migrate radially from the attached spheroids (Figure 5G).

### 3.4. Transplantation of EKC and HFB Spheroids Effectively Promotes Wound Closure and Epidermal Regeneration in Immunodeficient Mice

**Figure 6:**
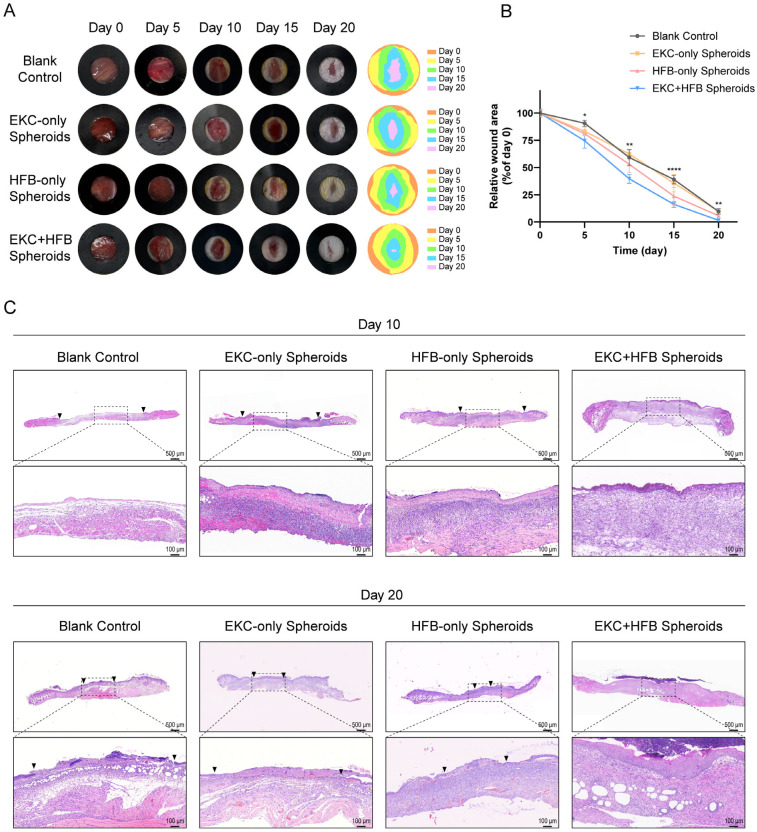
In vivo transplantation of EKC–HFB spheroids promotes wound healing. (**A**) Representative images illustrating the wound healing progression in different treatment groups over time (n = 5 independent samples per group). (**B**) Quantification of relative wound area normalized to day 0 at different time points. (n = 5 independent samples per group. Data are presented as mean ± SD, * *p* < 0.05, ** *p* < 0.01, **** *p* < 0.0001.) (**C**) Representative H&E staining images of wound samples from various treatment groups at Day 10 and Day 20. Arrow indicates epidermal edge. Scale bar = 500 μm (**upper**), 100 μm (**lower**).

**Figure 7:**
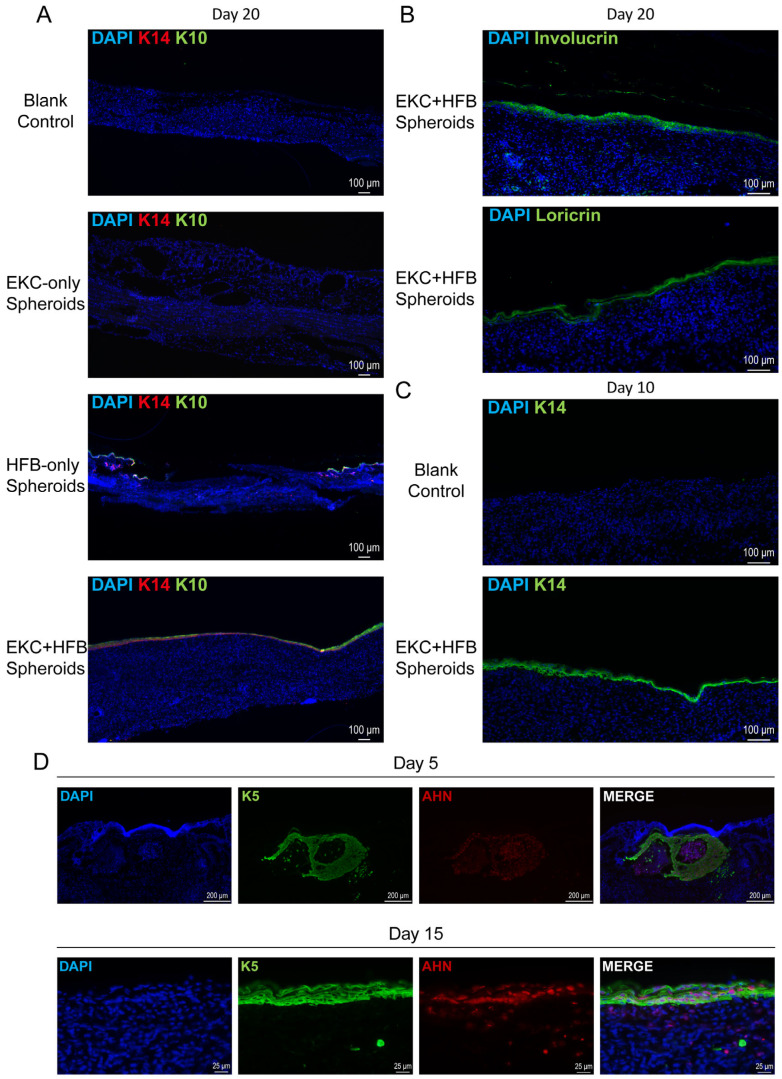
Epithelial regeneration and EKCs fate following EKC–HFB spheroids transplantation. (**A**) Representative immunofluorescence staining images in wound samples from different treatment groups at day 20. Scale bar = 100 μm. (**B**) Representative immunofluorescence staining images for maturation markers in wound samples from the EKC–HFB spheroids treatment group at day 20. Scale bar = 100 μm. (**C**) Representative immunofluorescence staining images in wound samples from blank group and EKC–HFB spheroid treatment group at day 10. Scale bar = 100 μm. (**D**) Representative immunofluorescence staining images of wound samples from the EKC–HFB spheroids treatment group at day 5 and day 15, demonstrating cell survival and integration. Scale bar = 200 μm (**upper**), 25 μm (**lower**).

To evaluate whether the hESC-derived EKCs could functionally accelerate wound closure and epidermal regeneration in vivo, we performed transplantation experiments using mixed cell spheroids in immunodeficient *mice*. Wound beds were treated with a small volume of culture medium (blank control, to model spontaneous healing), EKC spheroids alone, HFB spheroids alone, or combined EKC–HFB (3:1) spheroids. Among all treatment groups, the EKC–HFB spheroid group achieved the most rapid wound closure, with residual wound areas showing statistically significant differences compared to the blank control group at every time point examined. Treatment with the HFB-only spheroid group also enhanced healing compared to the blank control, particularly during the later phase. However, the EKC-only spheroid group did not significantly accelerate wound healing relative to the blank control, which will be explored further in the discussion. Notably, the EKC–HFB spheroid group consistently healed faster than the HFB-only spheroid group, with pronounced differences observed prior to day 10 (Figure 6A,B). H&E staining at day 10 post-wounding showed that only the EKC–HFB spheroid group had formed a continuous epidermis, despite a looser keratinocyte arrangement compared to native skin. No wound closure was observed in the other groups at this stage (Figure 6C, upper panel). By day 20, the EKC–HFB spheroid group had generated a complete skin structure with a well-stratified epidermis. However, all other treatment groups still presented with non-epithelialized regions, with the blank control and EKC-only spheroid group exhibiting larger unhealed areas than the HFB-only spheroid group (Figure 6C, lower panel). At day 20 post-wounding, immunostaining showed robust and continuous K14/K10 expression throughout the wound site in the EKC–HFB spheroid group. In the HFB-only group, positive staining for both markers was limited to the wound edges. However, no epithelialized areas were observed in the blank control or EKC-only groups within the corresponding fields (Figure 7A). The maturation state of the regenerated epidermis was further assessed by immunostaining for involucrin and loricrin. In the EKC–HFB spheroid group at day 20, robust expression of both terminal differentiation markers was observed, indicating the formation of a well-differentiated epidermal layer (Figure 7B). H&E staining at day 10 post-wounding revealed the presence of continuous epidermal-like tissue in the EKC–HFB spheroid group, although it appeared loosely organized. To confirm its epidermal identity, immunofluorescence staining for K14 was performed. The results showed continuous K14 expression at the wound center in the EKC–HFB spheroid group at day 10, whereas no fluorescent signal was detected in the corresponding region of the control group (Figure 7C). To further investigate the fate and survival of transplanted cells, tissue samples from the EKC–HFB spheroid group were harvested on days 5 and 15 post-transplantation. Sections were co-stained for K5 (an EKCs marker) and AHN (a human-specific marker) in order to identify and track the engrafted human cells. At day 5 post-transplantation, the EKCs and HFBs within the spheroids began to separate, with EKCs initially maintaining a clustered morphology (Figure 7D, upper panel). By day 15, the EKCs had spread across the wound surface to form a pluristratified epithelial sheet, while the HFBs localized within the underlying dermal layer (Figure 7D, lower panel). Positive AHN staining definitively confirmed that the transplanted human EKCs survived at the wound site, successfully migrated into the superficial layer of the wound, and transitioned from a spheroidal to a sheet-like architecture.

## 4. Discussion

In contrast to previous approaches that utilized pluripotent stem cell-derived keratinocytes for transplantation—typically as single-cell suspensions or pre-cultured cell sheets—both methods present inherent limitations: suspension grafts suffer from low engraftment efficiency, while pre-constructed cell sheets require prolonged in vitro culture and technically demanding manipulation. Our proposed EKC–HFB spheroid system overcomes these drawbacks. The spheroids self-assemble within 24 h, dramatically simplifying preparation procedures while enhancing post-transplantation cell survival. Furthermore, compared to current 3D organoid technologies—which are primarily employed for in vitro skin modeling and require extended culture periods—our strategy involves initial 2D differentiation followed by rapid 3D spheroid assembly. This approach allows for pre-differentiation and cryopreservation of cells, circumventing the need for protracted culture and demonstrating clear therapeutic efficacy in vivo. Furthermore, utilizing hESCs—a pluripotent stem cell source—for epidermal reconstruction establishes a critical foundation for advancing cell therapies toward clinical application.

In the present study, we successfully developed a strategy to generate 3D composite spheroids by co-culturing human embryonic stem cell-derived keratinocytes and human dermal fibroblasts, and demonstrated their efficacy in accelerating wound healing in immunodeficient *mice*. Validation of the pluripotent status of pluripotent stem cells serves as the fundamental basis for their directed differentiation in vitro [27,28]. Our characterization of the initial hESCs confirmed their robust pluripotency and genomic stability, providing a high-quality starting point for directed differentiation. Our induction protocol is stable and efficient, utilizing physiological selection through Collagen IV adherence to ensure a high-purity epidermal lineage and validated through flow cytometric analysis. While our differentiated EKCs showed lower expression of the maturation markers K5 and K14 than primary human epidermal keratinocytes, this finding reflects fundamental differences in population composition. Primary keratinocytes are a heterogeneous mix of cells at different differentiation stages—from stem cells to terminally differentiated cells—resulting in higher average K5/K14 expression. Importantly, our differentiated EKCs constitute a more homogeneous population with enhanced stemness relative to primary cells, representing a distinct advantage for cell treatment applications. A critical hallmark of functional keratinocytes is their ability to undergo maturation and form a stratified epithelium [29,30]. Our results from the air–liquid interface culture demonstrated that EKCs possess the intrinsic capacity to reconstruct a multilayered epidermal architecture that resembles native skin physiology [31]. Moreover, the stratified epidermal tissue expressed markers of mature epidermis, confirming its functionally differentiated state. Although the structural integrity and stratification speed of EKC-derived epidermis did not perfectly match those of primary human keratinocytes within the same timeframe, this discrepancy also reflects the inherent maturity gap between hESC-derived cells and their primary counterparts—a common observation in stem-cell-based regeneration [29,32,33]. Nevertheless, the successful formation of desmosomes and specific marker distribution underscores the functional potential of EKCs for skin reconstruction [34].

The core strategy of this study lies in the 3D co-assembly of EKCs and HFBs into biomimetic units to recapitulate the physiological epithelial–mesenchymal interaction. As the primary stromal component of the dermis, HFBs provide not only essential mechanical support but also vital biochemical cues through direct cell–cell contact and paracrine signaling [35], which are indispensable for maintaining the survival, proliferation, and differentiation potential of EKCs. This biomimetic design allows the spheroids to function as “active seeds” that can rapidly integrate into the wound bed and initiate the regenerative program upon transplantation.

**Figure A4:**
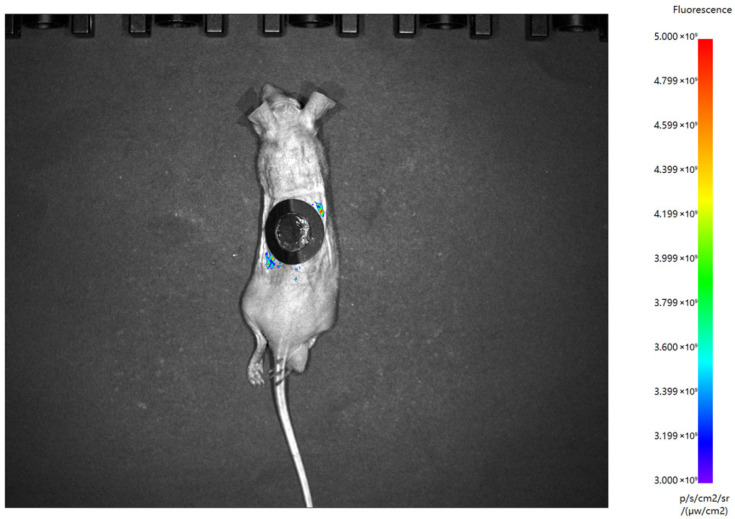
In vivo imaging of DiR-labeled EKCs transplanted into nude mice at day 3.

Compared to traditional single-cell suspension transplantation, the 3D spheroid configuration offers several distinct advantages [36,37]. First, the tissue-like microarchitecture—characterized by tight junctions, gap junctions, and cell–matrix interactions—significantly mitigates cellular stress and apoptosis during the harvesting and delivery process, facilitating rapid adaptation to the wound environment. Second, the relatively large physical size of the spheroids enhances their retention at the injury site, preventing them from being washed away by wound exudates or blood flow. In preliminary experiments, we attempted to track EKCs transplanted into nude *mice* using DiR-labeled cell suspensions via in vivo imaging. As shown in Figure A4, no fluorescent signal from the labeled EKCs was detected at the wound site by day 3 post-transplantation. We reasoned that the majority of cells in the suspension were lost due to immediate physical loss from the wound surface. The few residual cells likely succumbed to nutrient deprivation, osmotic stress, and the inflammatory wound microenvironment, ultimately leading to the absence of detectable fluorescence. This finding directly motivated our subsequent development of EKC–HFB spheroids. Overall, by mimicking the spatial arrangement and interaction network of native skin, spheroid transplantation exhibits superior therapeutic potential compared to dispersed cell delivery. We attribute this to the fact that other methods struggle to maintain high cell density and viability post-implantation; our 3D spheroids provide a pre-established niche that delivers a higher concentration of functionally primed cells per unit area.

However, the diffusion limits of nutrients and oxygen within 3D structures remain a significant technical consideration. To minimize core necrosis caused by insufficient perfusion, we optimized the fabrication process to maintain the spheroid diameter within a range (approximately 50,000 cells) that ensures effective mass exchange. Histological analysis revealed that the core of these spheroids is primarily composed of HFBs, which serve a supportive role; thus, localized central apoptosis has a negligible impact on the EKC-driven re-epithelialization process.

It should be noted that because our primary objective was in vivo transplantation, the in vitro observation period for the spheroids was relatively brief. Pathological analysis of the spheroids was conducted around day 5 post-assembly, which may explain why distinct stratification of EKCs was not yet prominent within the spheroids themselves at that stage [38]. Additionally, our animal experiments suggested that the spatial distribution and seeding density of the spheroids might influence the kinetics of epidermal reconstruction, which will be a focus of our future investigations.

In our in vivo experiments, the EKC-only group showed minimal therapeutic effect on wound healing. As shown in Figure 5A, we attribute this to the poor spheroid-forming capacity of EKCs due to their limited ability to secrete extracellular matrix. The resulting structures were mostly loose aggregates of a small number of cells, which were easily dissociated into single cells during transfer. Consequently, these transplanted cells exhibited limitations similar to those of single-cell suspensions—substantial cell loss or death—leading to negligible therapeutic outcomes. In contrast, while the HFB-only group failed to achieve early wound closure due to the absence of epidermal cells, it still promoted wound healing to some extent, particularly during the later stages. This delayed effect may be attributed to extracellular matrix deposition and growth factor secretion by fibroblasts, which could support tissue remodeling and regeneration [39]. Interestingly, we observed that *mice* receiving EKC–HFB spheroid transplants achieved rapid wound closure through the formation of a continuous, albeit fragile, epidermal layer even before the full maturation of the underlying granulation tissue. This accelerated barrier restoration is one of the most clinically significant outcomes of epidermal cell therapy. We also noted the presence of non-human-derived epidermis adjacent to the transplant sites, suggesting that the engrafted cells may exert a chemoattractive effect on host epithelial cells, thereby stimulating endogenous repair. While our results confirmed the efficacy of this approach in accelerating wound closure, the observation period was limited to the early stages of healing. Further studies are warranted to evaluate the long-term quality of wound repair, including the extent of scar reduction, the promotion of vascularization, the regeneration of skin appendages, and the functional integrity of the reconstructed epidermis.

Our study has several limitations. First, to validate the transplantation method and obtain standardized results, we utilized a commercially available human fibroblast cell line. In future studies, we aim to employ hESC-derived fibroblasts to construct more consistent cell transplantation units. This approach would more fully leverage the advantages of hESC pluripotency and definitively address the issue of cell source availability. It will also facilitate clinical translation. Furthermore, immunogenicity remains a critical challenge that must be addressed prior to clinical translation. While our proof-of-concept studies in immunodeficient *mice* successfully demonstrated the therapeutic potential of EKC–HFB spheroids, we acknowledge that immunogenicity remains a critical barrier to clinical translation. The transplantation of allogeneic hESC-derived cells into immunocompetent recipients would likely elicit immune rejection due to HLA mismatching between donor cells and the host [40]. To address these challenges, multiple strategies have been proposed and warrant future investigation in the context of our cell therapy approach. One promising avenue is the establishment of HLA-matched cell banks representing common HLA haplotypes, which would reduce but not eliminate the need for immunosuppression. Furthermore, recent advances in genome editing have enabled the generation of hypoimmunogenic universal donor cells through targeted modifications [41]. For instance, disruption of HLA class I and II molecules via CRISPR/Cas 9 has shown potential to create pluripotent stem cells with reduced immunogenicity [42,43]. Alternatively, encapsulation strategies using immunoprotective biomaterials could physically shield transplanted cells from host immune cells while allowing nutrient and waste exchange. It has also been suggested that hESC-derived epidermal cells possess intrinsically lower immunogenicity, provoking less immune rejection [44]. Accordingly, the development of hypoimmunogenic seed cells offers another avenue for addressing immune barriers.

Furthermore, for human embryonic stem cell-based therapies to achieve true clinical application, current Good Manufacturing Practice (cGMP) compliance must also be taken into consideration [21,27,45,46]. First, the use of human embryonic stem cells necessitates rigorous ethical oversight, including confirmation that the original cell lines were derived in accordance with informed consent and applicable ethical guidelines. Second, for clinical application, the differentiation protocol must be adapted to comply with cGMP standards to ensure the safety, purity, and consistency of the final cell product. Third, a critical safety concern is the potential presence of residual undifferentiated hESCs, which could lead to teratoma formation. Therefore, comprehensive quality control measures—such as in vivo tumorigenicity assays—would be required prior to clinical use.

And it is important to note that the differentiation efficiency and characteristics of keratinocytes derived from pluripotent stem cells may be cell-line-dependent. Our current study was primarily conducted using the H9 human embryonic stem cell line. While H9 is a well-characterized and widely used cell line in research, the results obtained may not be directly translatable to other cell lines. Future studies should validate these findings using cGMP-grade cell lines to assess the robustness and reproducibility of our differentiation protocol under conditions relevant for translational medicine.

Future studies should focus on both validating our findings using cGMP-compliant pluripotent stem cell lines and developing approaches to reduce immune rejection, followed by regulatory-compliant preclinical safety and efficacy studies to facilitate eventual clinical translation.

## 5. Conclusions

In this study, we successfully developed a robust skin regeneration strategy based on hESCs. We first achieved the directed differentiation of hESCs into keratinocytes and demonstrated their significant proliferative capacity and potential to form stratified epidermal structures. By co-assembling EKCs with human dermal fibroblasts, we engineered highly bioactive and biomimetic 3D composite spheroids. In an immunodeficient mouse model, the transplantation of these spheroids significantly accelerated the healing of full-thickness skin defects and effectively reconstructed a functional epidermal barrier. The successful integration of human-derived cells and the observation of epidermal stratification further confirm the efficacy of this strategy in promoting tissue regeneration in vivo. This 3D spheroid-based transplantation modality addresses the clinical shortage of donor skin by leveraging the near-infinite expansion potential of hESCs. The standardized fabrication process established in this work—characterized by its simplicity, short preparation cycle, and scalability—offers a promising new avenue for clinical translation in skin tissue engineering and regenerative medicine.

## Data Availability

The experimental data designed in this study can be obtained from Mendeley Data at https://doi.org/10.17632/79p4d457rb.1 (accessed on 24 March 2026).

## Abbreviations

The following abbreviations are used in this manuscript:

hESCs	human embryonic stem cells
EKCs	human-embryonic-stem-cell-derived keratinocytes
HFBs	human dermal fibroblasts
ECM	extracellular matrix
TGF-β	transforming growth factor-beta
FGF	fibroblast growth factor
BMPs	bone morphogenetic proteins
KCs	keratinocytes
EMI	epithelial–mesenchymal interaction
K18	keratin 18
K5	keratin 5
K14	keratin 14
K19	keratin 19
K10	keratin 10
TEM	transmission electron microscopy
PDT	population doubling time
ALI	air-liquid interface
H&E staining	hematoxylin and eosin staining
AHN antibody	anti-human nuclei antibody
DMEM	Dulbecco’s Modified Eagle Medium
FBS	fetal bovine serum
cGMP	current Good Manufacturing Practice
